# Transplantation of Normal Adipose Tissue Improves Blood Flow and Reduces Inflammation in High Fat Fed Mice With Hindlimb Ischemia

**DOI:** 10.3389/fphys.2018.00197

**Published:** 2018-03-08

**Authors:** Liyuan Chen, Lin Wang, Yongjie Li, Liqun Wuang, Yaofang Liu, Ningbo Pang, Yulin Luo, Jing He, Liping Zhang, Ni Chen, Rong Li, Jianbo Wu

**Affiliations:** ^1^Drug Discovery Research Center, Southwest Medical University, Luzhou, China; ^2^Laboratory for Cardiovascular Pharmacology of Department of Pharmacology, The School of Pharmacy, Southwest Medical University, Luzhou, China; ^3^Department of Gynaecology and Obstetrics, The Affiliated Hospital of Southwest Medical University, Luzhou, China; ^4^Dalton Cardiovascular Research Center, University of Missouri, Columbia, MO, United States

**Keywords:** arteriogenesis, inflammation, adipose tissue, blood perfusion, high-fat diet

## Abstract

**Background:** Fat deposition is associated with peripheral arterial disease. Adipose tissue has recently been implicated in vascular remodeling and angiogenic activity. We hypothesized that the transplantation of adipose tissues from normal mice improves blood flow perfusion and neovascularization in high-fat diet fed mice.

**Methods:** After 14 weeks of high-fat diet (HFD)-fed mice, unilateral hind limb ischemia was performed. Subcutaneous white adipose tissue (WAT) and brown adipose tissue (BAT) fat pads were harvested from normal EGFP mice, and subcutaneously transplanted over the region of the adductor muscles of HFD mice. Blood flow was measured using Laser Doppler Scanner. Vascular density, macrophages infiltration, and macrophage polarization were examined by RT-qPCR, and immunohistochemistry.

**Results:** We found that the transplantation of WAT derived from normal mice improved functional blood flow in HFD-fed mice compared to mice transplanted with BAT and sham-treated mice. WAT transplantation increased the recruitment of pericytes associated with nascent blood vessels, but did not affect capillary formation. Furthermore, transplantation of WAT ameliorated HFD-induced insulin resistance, M2 macrophage predominance and the release of arteriogenic factors in ischemic muscles. Mice receiving WAT also displayed a marked reduction in several proinflammatory cytokines. In contrast, mice transplanted with BAT were glucose intolerant and demonstrated increased IL-6 levels in ischemic muscles.

**Conclusion:** These results indicate that transplantation of adipose tissue elicits improvements in blood perfusion and beneficial effects on systemic glucose homeostasis and could be a promising therapeutic option for the treatment of diabetic peripheral arterial disease.

## Introduction

The distribution of adipose tissue plays a fundamental role in the prevalence of obesity-related comorbidities. Obesity has been implicated in the development or progression of a wide variety of disorders, including hypertension, type II diabetes mellitus, and dyslipidemia, which could cause peripheral arterial disease (PAD) (Criqui and Aboyans, 2015). Accumulating evidence has demonstrated that adipose tissue (AT) distribution is associated with insulin resistance and type 2 diabetes (Gautier et al., 1998; Cnop et al., 2002; Azuma et al., 2007). Furthermore, recent reports in animal model and human samples suggest that adipose tissue accumulation imparts distinct angiogenic activity, i.e., the percentage of visceral adipose tissue explants developing capillary branches was lower than those from subcutaneous adipose tissue, leading to an impaired angiogenic response (Pasarica et al., 2009; Gealekman et al., 2011). Capillary sprouting from adipose tissue explants reflects the ability of adipose cells to proliferate, migrate and interact with vascular structures (Tran et al., 2012), suggesting that adipocytes can develop *in vivo* from cells of endothelial origin.

It has been reported that central obesity is associated with PAD, suggesting that ectopic distribution of adipose tissue may be linked to lower-extremity arterial diseases (Planas et al., 2001; Fox et al., 2010). In humans and other mammals, adipose tissue can be classified into two subtypes with opposing functions: white adipose tissue (WAT) and brown adipose tissue (BAT). BAT contains a large number of mitochondria that act to increase energy expenditure, whereas the main function of WAT is to store triglycerides. Thus, regulatory factors related to adipose distribution could be clinically useful for the treatment of obesity. In lean subjects, adipose tissue macrophages (ATMs) contribute to the regulation of adipose tissue function as well as angiogenesis (Cho et al., 2007; Nishimura et al., 2007; Bourlier et al., 2008). In addition to adipocytes, WAT contains macrophages, leukocytes, fibroblasts, and endothelial cells, all of which are a rich source of cytokines and growth factors. Adipose tissue macrophages could be potential mediators of the formation of collateral circulation. Transplantation of both white and brown adipose tissue produces different beneficial effects associated with the regulation of glucose homeostasis and insulin sensitivity (Thomou et al., 2007; Tran et al., 2008). In particular, transplantation of subcutaneous AT can improve glucose homeostasis via endocrine effects in HFD-fed mice (Hocking et al., 2015). Recently, Min et al. (2016) demonstrated the potential of capillary progenitors to cure obesity-associated disturbances in glucose homeostasis ATMs consist of two different phenotypes (i.e., classically activated M1 macrophages and alternatively activated M2 macrophages. Previous studies proposed that M1 ATMs display the CD11c surface marker, and produce proinflammatory cytokines, such as tumor necrosis factor (TNF)-α, interleukin (IL)-6, and monocyte chemoattractant protein (MCP)-1, thus leading to the induction of insulin resistance (Lumeng et al., 2007a,b; Fujisaka et al., 2009). On the other hand, M2 ATMs, are characterized by the expression of CD206, transforming growth factor (TGF)-β1, fibronectin 1 (Fn1), and IL-10, which are involved in the remodeling of tissues (Lumeng et al., 2007a,b; Guglielmi et al., 2015). Macrophage infiltration of WAT leads to increased lipolysis through the increased release of interleukin-6 and other macrophage-derived cytokines. Furthermore, periadventitial fat has been primarily considered to act as a structural support for blood vessels. It is unclear whether adipose tissue cellular remodeling related to chronic inflammation and vascular alterations may compromise surrounding blood vessel function and negatively affect glucose homeostasis in peripherally ischemic type 2 diabetic mice. We investigated the effect of adipose tissue on the ischemia-induced neovasculature and on the development of inflammation in HFD-induced critical limb ischemia after vascular injury. We demonstrated that mouse subcutaneous adipose tissue can improve ischemic limb blood perfusionin HFD-fed mice.

## Materials and methods

### Animals

C57BL/6J mice were from sourced from the Chongqing Medical University Animal Center, Chongqing, China. All protocols for animal use were reviewed and approved by the Animal Care Committee of Southwest Medical University in accordance with Institutional Animal Care and Use Committee guidelines.

### High fat diet-fed mouse model

Four- to five-week-old male C57BL/6J mice were fed a high-fat diet (HFD; 45% fat by kcal) (D12451; Research Diet, New Brunswick, NJ) for 14 weeks, as described previously (Hazarika et al., 2007). Age-matched male mice fed a normal diet (ND) served as controls. Blood glucose levels were measured from tail vein blood samples using an automatic glucometer (Accu-Check; Roche Diagnostics, Mannheim, Germany).

Body weight was monitored every 3 days. At the completion of the study 21 days after transplantation, blood was collected from the inferior vena cava using a 1-mL syringe and centrifuged at 1,500 × g for 10 min to measure fasting serum glucose, low-density lipoprotein (LDL), high-density lipoprotein (HDL), total cholesterol (TC), and triglycerides (TG).

### Mouse hindlimb ischemia model

Unilateral hindlimb ischemia was induced in mice by ligation and excision of a segment of the left femoral artery, as previously described (Wu et al., 2015). Mice were anesthetized using intraperitoneal sodium pentobarbital (60 mg/kg body weight) or isoflurane (5% by inhalation). A subcutaneous dose of buprenorphine hydrochloride (0.1 mg/kg) was administered for analgesia. Additional sodium pentobarbital (12 mg/kg body weight) or 5% isoflurane was given as needed to maintain anesthesia. Theleft femoral artery was exposed, ligated proximally and distally with 5-0 silk ligatures, and the femoral bifurcation with all branches was excised. Mice were euthanized by cervical dislocation at the end of the experiment while still under anesthesia. Perfusion of the ischemic and non-ischemic hindlimb was measured in each mouse by laser-Doppler imaging (LDI) immediately before surgery, immediately after ligation, and at 3, 7, 11, 14, and 21 days after ligation using a scanning moorLDI2-HIR (Moor Instruments, Wilmington, Del) high-resolution laser Doppler imager.

### Adipose tissue transplantation

For the transplantation experiments, subcutaneous WAT and BAT fat pads were harvested from 8-week-old male enhanced Green Fluorescent Protein (eGFP) mice (background strain of C57BL/6J), cut into one 50-mg piece and subcutaneously transplanted over the region of the adductor muscles of 20-week-old recipient HFD mice fed a high fat diet for 14 weeks (*n* = 9 male mice per group). Each recipient HFD mice received an equivalent transplanted fat mass. Transplanted mice underwent surgery by ligation and excision of a segment of the left femoral artery, as described above. Finally, the musculofascial and skin incisions were sutured. Sham surgeries of control animals were performed in the same manner, but without fat pad transplantation. Three weeks after transplantation, the flow perfusion of the hindlimb was evaluated.

#### *In vivo* optical imaging

Anesthetized mice were placed in an *in vivo* FX PRO (BRUKER Corporation, Billerica, MA, USA). Scanning was performed on the transplanted fat of mice. Fluorescent images were recorded using two-step scan (GFP 30s plus white light 0.175s) at 3, 7, 10, 14, and 21 days after ligation. eGFP was obtained with an excitation wavelength of 480 ± 10 nm and emission wavelength of 510 ± 10 nm.

### Quantitative real-time PCR

Hindlimb muscles were collected at baseline or 21 days post-ischemia and RNA was extracted using TRIzol reagent (Invitrogen, Carlsbad, CA, USA). RNA was pre-treated with deoxyribonuclease I (Invitrogen Life Technologies, Carlsbad, CA, USA), and a SuperScript kit (Invitrogen Life Technologies, Carlsbad, CA, USA) was used to synthesize cDNA according to the manufacturer's recommendations. Each sample was analyzed in duplicate with ribosomal 18S RNA as an internal control. All fold changes in gene expression were determined using the 2–ΔΔCT method. The values are presented as the mean ± SEM. All primers are listed in Supplemental Table 1.

### Glucose and insulin tolerance testing

After an overnight fast, a glucose tolerance test (GTT) was performed following an intraperitoneal (IP) injection of D-glucose (Roth, Karlsruhe, Germany) (2 g of glucose/kg body mass). Insulin tolerance testing was performed using IP injections of insulin (0.75 U insulin/kg body mass) after a 4-h fast. Blood samples were then obtained from the caudal vein, and the blood glucose level was measured 0, 30, 60, and 120 min after glucose injection using a One Touch® Vita® glucometer (Zug, Switzerland).

### Histological assessment

Mouse adductor and gastrocnemius muscles were obtained 3 weeks after hind limb ischemia surgery and fixed with 4% (wt/vol) paraformaldehyde in PBS for 3 h and transferred to 30% (wt/vol) sucrose overnight. Then, the samples were embedded in OCT compound, frozen, and serially sectioned (6 μm). Cross-sections were prepared for immunofluorescence analysis. Capillary density, vascular smooth muscle cells/pericytes, and macrophages were determined by immunostaining using anti-PECAM-1 (Santa Cruz Biotechnology Inc., Santa Cruz, CA, USA), anti-NG2 (Santa Cruz Biotechnology Inc., Santa Cruz, CA, USA), and anti-F4/80 (Abcam, Cambridge, UK) antibodies, respectively. The secondary antibodies were goat anti-rabbit IgG Alexa Fluor 568-conjugated antibodies (Molecular Probes, Invitrogen). Images were captured with a fluorescence microscope (Leica). Numbers were quantified in 5 microscopic fields in each of 3 cross-sections of each implant using ImagePro Plus software.

### Statistical analysis

All data are presented as the mean ± SEM. Glucose excursions during the GTT were calculated using Microsoft Excel and expressed as AUC. Differences between groups were analyzed by Student's *t*-test (comparisons of two groups) or analysis of variance (ANOVA; multiple comparisons) using GraphPad Prism (La Jolla, CA, USA). *P* < 0.05 was considered to represent statistical significance.

## Results

### Transplantation of adipose tissues

**Figure 1:**
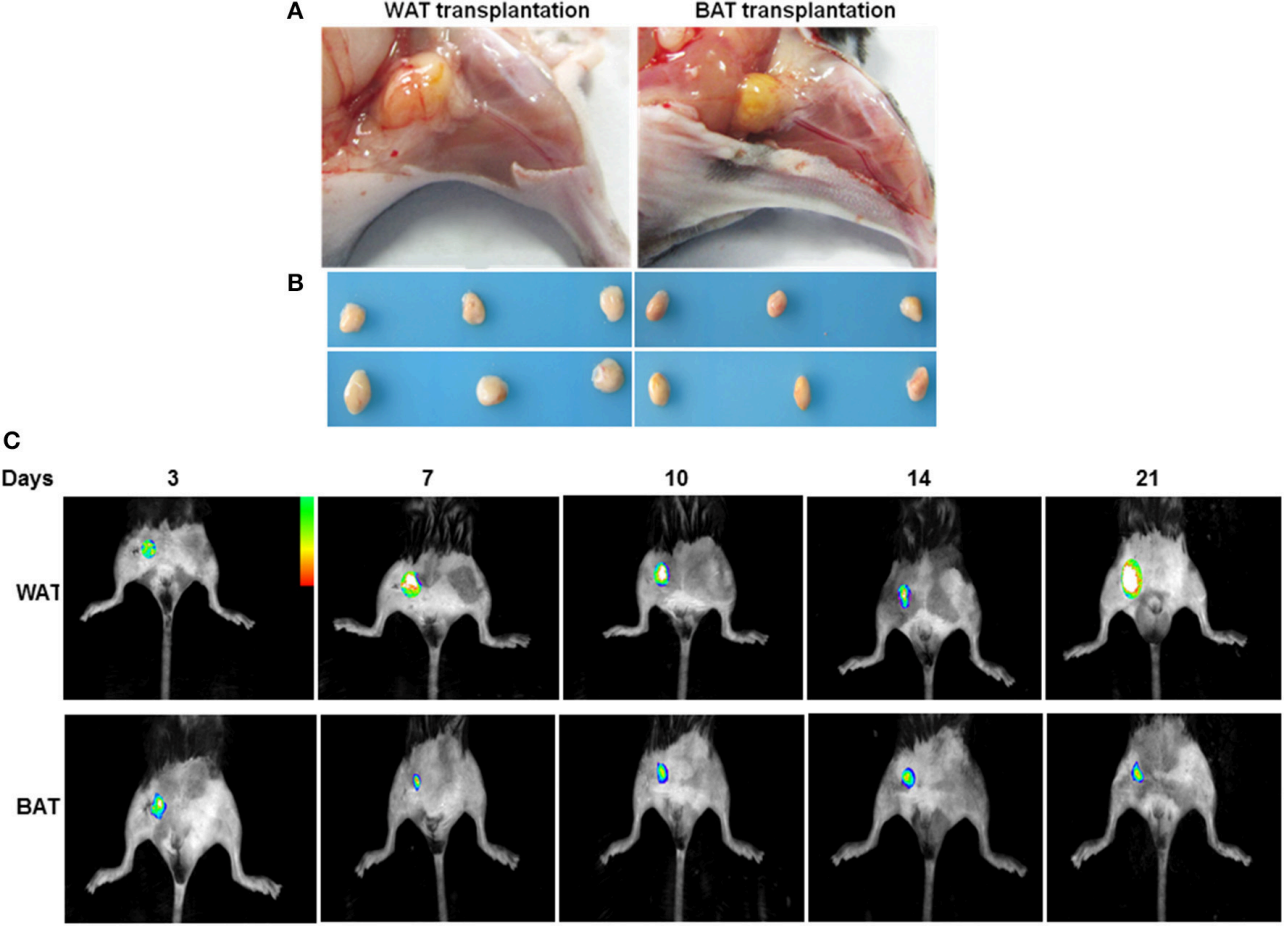
Adipose tissues 21 days after transplantation. **(A)** The depot (yellow, at center, originally 50 mg) subcutaneously transplanted over the adductor muscle region. **(B)** Adipose tissue depots 21 days after transplantation. Representative images of transplanted AT at the completion of the treatment protocol. **(C)** Transplanted eGFP pads were imaged by capturing fluorescence signal using an *in-vivo* Imaging System at indicated time points.

To examine the significance of adipose tissue transplantation in a pathological model relevant to human cardiovascular disease, we fed mice high-fat chow for 14 weeks to induce obesity and hyperglycemia. To examine the role of adipose tissues in reversing blood perfusion in HFD mice, we introduced wild-type fat by surgical implantation. A subcutaneous WAT and BAT fat pad were harvested from 8-week-old male eGFP mice and cut into 50-mg pieces. They were then subcutaneously transplanted over the adductor muscle region of recipient HFD mice. At 21 days after transplantation, the grafts had a healthy gross and morphological appearance that was visible by fluorescent imaging (Figures 1A–C). eGFP expressing cells were observed in the sections of ischemic adductor muscles in both WAT and BAT transplanted mice (Supplemental Figure 1). By 21 days after transplantation in all subject groups, there was no significant difference in the total weight between WAT transplantation (27.8 ± 0.58 g), BAT transplantation (28.2 ± 0.61 g) and sham-operated mice (28.2 ± 0.36 g) (Supplemental Figure 2). For serum lipids, LDL, HDL, TC, and TG, no significant differences were found between all groups (Supplemental Table 2). Finally, there were no changes in the circulating levels of TNF-α and SDF-1α in mice receiving transplants of WAT compared with mice receiving BAT transplants or sham-operated mice (Supplemental Table 2).

### WAT transplantation reverses HFD-impaired blood flow perfusion

**Figure 2:**
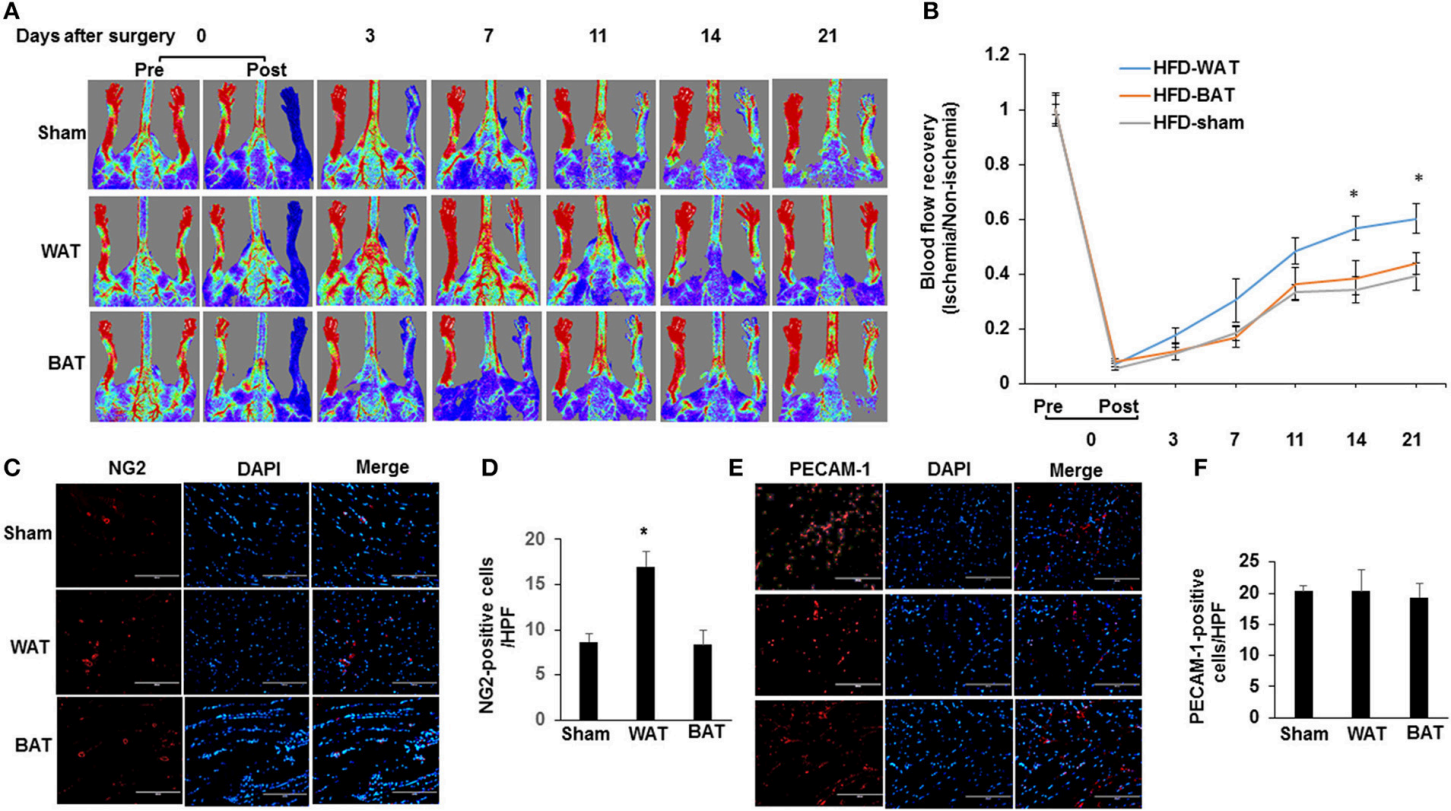
WAT transplantation reverses HFD-impaired blood flow perfusion after femoral artery ligation. **(A)** Representative laser Doppler images of mouse hindlimbs at indicated time points after femoral artery ligation. Pre and post indicate immediately before and after surgery, respectively. Red color denotes normal perfusion. **(B)** Mean ratio of blood flow in ischemic and non-ischemic hindlimb foot pads for all animals at indicated the time points (*n* = 9 per group; ^*^P < 0.05 vs. BAT-transplanted and sham mice). **(C)** Representative images of arterioles as assessed by anti-NG2 immunostaining in ischemic adductor muscles 21 days after femoral artery interruption. Distance bars, 100 μm. **(D)** The mean arteriole density in ischemic adductor muscles was significantly greater in WAT-transplanted mice (*n* = 9 per group; ^*^P < 0.05 vs. BAT-transplanted and sham mice). **(E,F)** Representative images of capillary as assessed by anti-PECAM-1 immunostaining in ischemic gastrocnemius muscles 21 days after femoral artery interruption. Distance bars, 100 μm. Mean capillary density in ischemic gastrocnemius muscle did not differ significantly between groups (*P* > 0.5). HPF indicates high-power field.

To determine whether AT transplantation mediates the formation of functional vasculature, hindlimb ischemia was induced in HFD-fed mice by ligation and excision of the femoral artery, after which, mice received either eGFP-derived WAT, BAT, or a sham vehicle control. After 21 days of transplantation, laser Doppler imaging revealed that the recovery of perfusion in the ischemic hindlimb was greater in WAT-transplanted mice compared to either BAT-transplanted or sham mice (Figures 2A,B). Interestingly, there was no difference in the blood perfusion between BAT-transplanted and sham mice. Consistent with these results, arteriole density in ischemic adductor muscles 21 days after induction of ischemia was significantly increased in WAT-transplanted mice vs. BAT-transplanted (17 ± 1.65 vs. 8.3 ± 1.57 NG2-positive cells/HPF; *p* = 0.015) and sham controls (17 ± 1.65 vs. 8.7 ± 0.95 NG2-positive cells/HPF; *p* = 0.009) (Figures 2C,D). However, capillary density in ischemic gastrocnemius muscles did not significantly differ between the experimental groups (Figures 2E,F). Taken together, our results demonstrate that implantation of WAT promoted collateral arteriole development and the recovery of blood perfusion in response to hindlimb ischemia.

### Macrophage quantification in ischemic muscles

**Figure 3:**
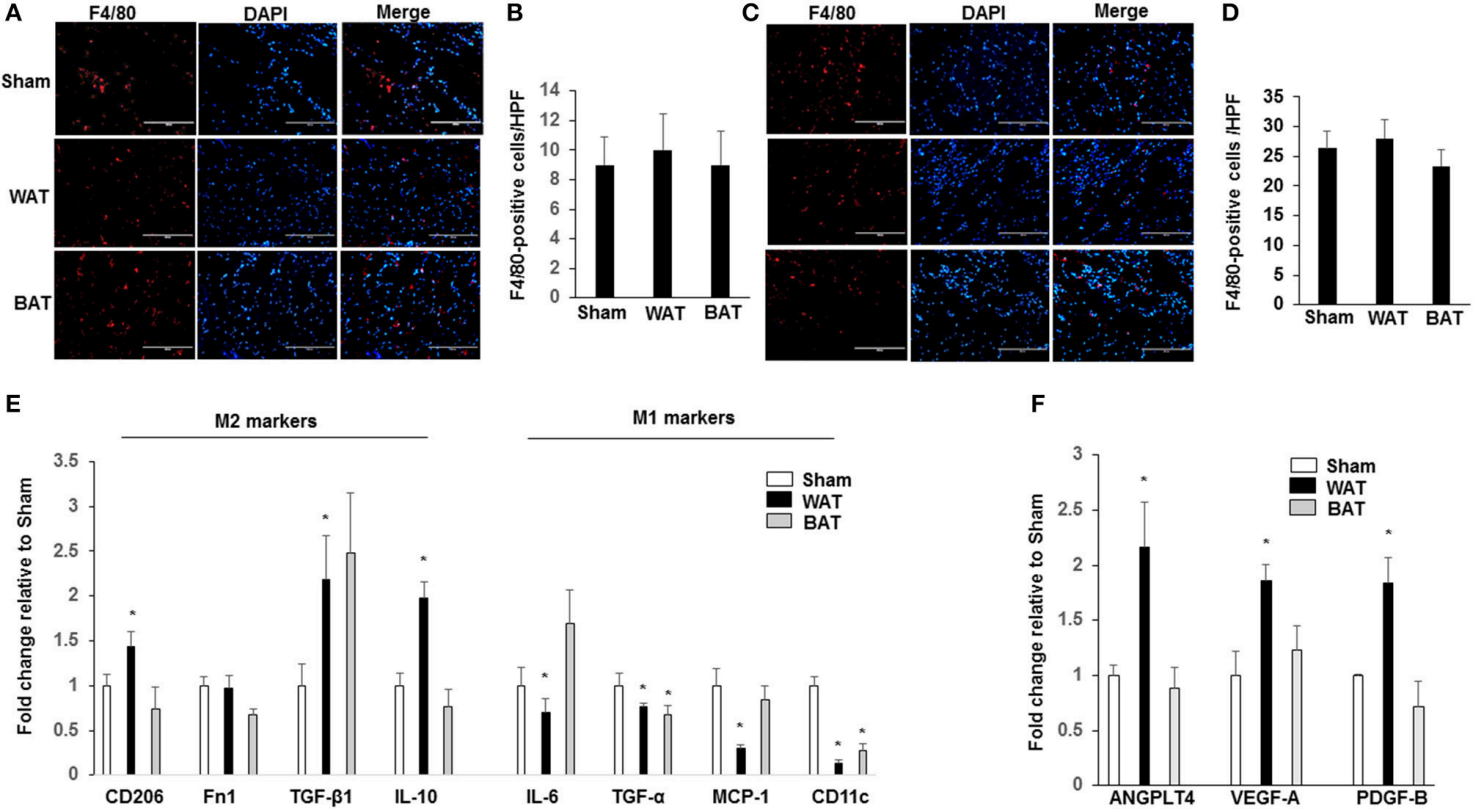
Macrophages display an M2-phenotype in ischemic muscles from transplanted WAT mice. Representative images of macrophages as assessed by F4/80 immunostaining, in ischemic adductor muscle **(A)** and gastrocnemius muscle **(C)** recovered 21 days after femoral artery interruption. Quantification of anti-F4/80 positive-macrophage infiltration of ischemic adductor muscle **(B)** and gastrocnemius muscle **(D)**. Scale bars, 100 μm. **(E)**. The gene profile of the M1- and M2-macrophage phenotype by quantitative RT-PCR of ischemic adductor muscles 21 days after surgery. **(F)** The gene analysis of ANGPTL4, VEGF-A, and PDGF-B by quantitative RT-PCR of ischemic adductor muscles 21 days after surgery. All bars show Mean ± SEM. Data are mean of triplicate experiments and are expressed as fold-control. ^*^P < 0.05 toward sham-operated mice.

We examined whether increased collateral arteriole density in WAT-transplanted ischemic muscle correlated with differences in macrophage number or with their polarization state by quantitative RT-PCR. We found that there was a significant increase in the total macrophage number (F4/80-positive) of subcutaneous WAT in HFD-WT mice compared with that in normal diet mice (Supplemental Figure 3). Importantly, there was no significant difference in the total macrophage number in both ischemic adductor (Figures 3A,B) and gastrocnemius muscles (Figures 3C,D) between WAT, BAT, and sham-treated mice. To investigate the effect of AT transplantation on the polarization of resident macrophages in ischemic muscles, transcript markers for the M1 and M2 macrophage phenotype were assessed by quantitative RT-PCR in ischemic adductor muscles. The expression levels of the M2 markers CD206 (1.44 ± 0.16; *p* < 0.05), TGF-β1 (2.18 ± 0.49; *p* < 0.05) and Fn1 (1.97 ± 0.19; *p* < 0.05) were significantly higher in ischemic muscles from transplanted WAT mice than either transplanted BAT or sham mice (Figure 3E). Correspondingly, proinflammatory M1 markers, including IL-6 (0.69 ± 0.16; *p* < 0.05), TNF-α (0.77 ± 0.03; *p* < 0.05), MCP-1 (0.30 ± 0.03; *p* < 0.05), and CD11c (0.13 ± 0.03; *p* < 0.05), were significantly lower in ischemic muscles from transplanted WAT mice compared with that from sham mice (Figure 3E).

Previous studies demonstrated that a recently identified secreted factor, angiopoietin-like protein 4 (ANGPTL4), is a proangiogenic factor secreted from AT. We further analyzed the mRNA levels of ANGPTL4, VEGF-A, and PDGF-B in ischemic muscles and found that the expression of ANGPTL4 (2.16 ± 04; *p* < 0.05), VEGF-A (1.86 ± 0.14; *p* < 0.05), and PDGF-B (1.84 ± 0.23; *p* < 0.05) was significantly increased in mice transplanted with WAT compared to mice transplanted with BAT and sham controls (Figure 3F).

### Transplantation of at improves glucose tolerance and insulin sensitivity

**Figure 4:**
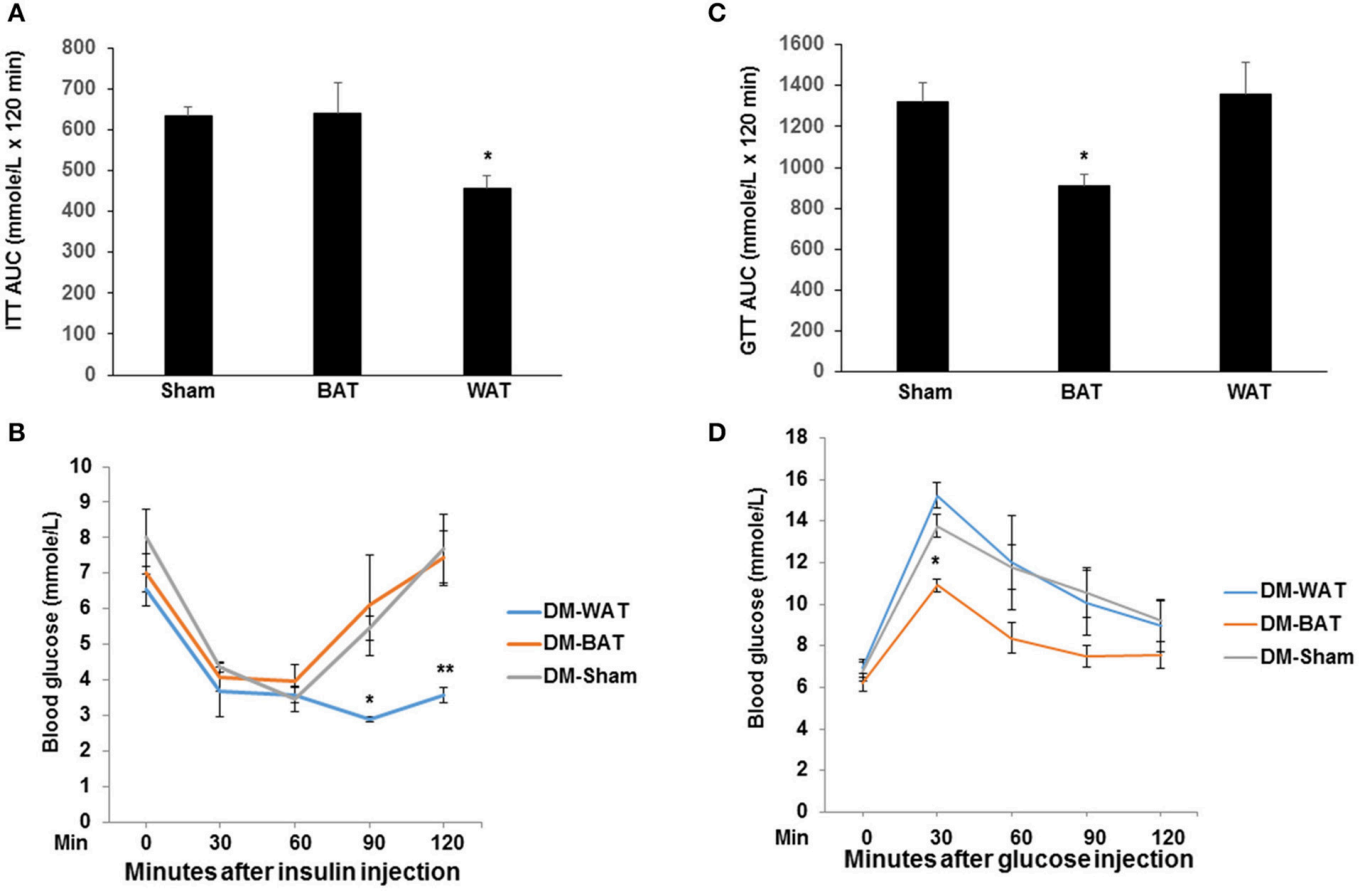
Transplantation of WAT improves systemic insulin sensitivity and glucose tolerance. Mice were fed NCD or HFD for 14 weeks, followed by transplantation of WAT, BAT or sham-operation for 21 days. **(A,B)** Insulin tolerance test (ITT) and AUC in each group. *n* = 6 per group. ^*^P < 0.05 vs. BAT-transplanted and sham-operated mice. **(C,D)** Glucose tolerance test (GTT) and AUC in the AT transplant HFD mice. *n* = 6 per group. ^*^P < 0.05 vs. WAT-transplanted and sham-operated mice.

Previous studies demonstrated that mouse subcutaneous adipose tissue can improve glucose homeostasis in HFD-fed mice. To directly investigate this possibility in our HFD-fed mice hindlimb ischemia model, we performed intraperitoneal insulin and glucose tolerance tests on mice implanted with AT and sham mice. By 21 days after WAT transplantation, there was a significant improvement in insulin tolerance compared to mice transplanted with BAT and sham-operated mice (Figures 4A,B). Similarly, we found that BAT transplanted mice ameliorated HFD-induced insulin resistance (Figures 4C,D). These results suggest that transplantation improves glucose tolerance and insulin sensitivity.

## Discussion

A series of studies revealed a regulatory relationship between AT and PAD (Planas et al., 2001; Fox et al., 2010). As an endocrine organ, AT could serve as a promising new potential therapeutic target for obesity and its related diseases. Previous reports demonstrated that AT releases cytokines (Nakagami et al., 2005) and growth factors that promote perivascular inflammation (Henrichot et al., 2005) and smooth muscle cell proliferation (Barandier et al., 2005). These findings suggest that AT may promote vascular disease by regulating inflammatory cells and vascular smooth muscle cells. Transplantation of AT has a beneficial effect on obesity in high-fat diet-induced obese mouse models (Tran et al., 2008; Hocking et al., 2015). In this study, we asked whether transplantation of AT might restore type-2 DM-mediated inhibition of ischemia-induced neovasculature. We also performed BAT transplantation in HFD-induced mice. In the present study, we demonstrated that WAT transplantation recovered blood perfusion in a HFD-induced diabetic limb ischemia mouse model. This is the first study to show that WAT transplantation results in the activation of M2 macrophage polarization in ischemic muscles, eventually leading to improved arteriogenesis and insulin homeostasis. In addition, BAT transplantation alleviated diet-induced glucose intolerance. The recruitment of inflammatory cells and macrophages in particular is responsible for arteriogenesis (collateral vessel remodeling) (Simons and Ware, 2003; Schaper, 2009). Recent studies have emphasized the complexity of *in vivo* macrophage polarization, which displays a continuum of diverse functional states from the microenvironment (Gordon et al., 2014; Xue et al., 2014). Previous reports suggested that the polarization of both types of macrophage is required during arteriogenesis and that the release of chemotactic factors, including TNF-α, IL-6 and MCP1 by proinflammatory M1 macrophages enhanced the recruitment of circulating monocytes (Arras et al., 1998; Troidl et al., 2013). However, most studies have demonstrated a more prominent role for anti-inflammatory M2 macrophages as they express a considerable number of angiogenic growth factors that promote arteriogenesis, including VEGF-A, SDF-1, PDGF-B, and HGF (Kodelja et al., 1997; Arnold et al., 2007; Nucera et al., 2011; Takeda et al., 2011). Furthermore, M2 macrophages were shown to promote arteriogenesis and enhance tissue protection during mouse hindlimb ischemia (Takeda et al., 2011).

Macrophage polarization yields different functional properties depending on the tissue microenvironment. There is evidence that adipose tissue plays a role in regulating the macrophage phenotype. Adipose tissue in obese and diabetic subjects has been shown to contain increased numbers of M1 macrophages, a major source of proinflammatory cytokines (Chawla et al., 2011). Recent studies examined whether transplantation of adipose tissue could affect activation of macrophages in recipient subjects. Indeed, mice receiving subcutaneous fat displayed a marked reduction in HFD-induced increases in the plasma concentrations of several proinflammatory cytokines compared to sham-operated mice (Hocking et al., 2015). In this study, we observed that the total macrophage infiltrate was not significantly different from ischemic muscles between WAT, BAT and sham-treated mice. We further found a significant reduction in a subset of M1 macrophage–specific genes in ischemic adductor muscles of WAT transplanted mice compared to BAT transplanted and sham mice, whereas M2 macrophage genes showed increased expression. We observed that there was a significant reduction in the level of TNF-α mRNA in ischemic muscles after WAT transplantation. On the other hand, our results indicated that IL-6 mRNA was significantly increased by BAT transplantation. These results imply that the transplantation of WAT and BAT into mice on a high fat diet confers differential beneficial effects on glucose metabolism.

ANGPTL4 is expressed at higher levels in human subcutaneous adipose tissue and has been identified as an adipokine that is involved in proangiogenesis and lipid metabolism (Gealekman et al., 2008, 2011). In addition to ANGPTL4 mRNA, we observed that VEGF-A and PDGF-B mRNA expression in ischemic muscles were significantly increased in mice transplanted with WAT compared with mice transplanted with BAT and sham controls. Thus, this difference between WAT and BAT could at least in part explain the observed difference in angiogenic capacity between these depots, and thus may represent an additional angiogenic mechanism utilized by WAT. Future studies should analyze the individual components released by adipocytes derived from transplanted AT under both physiological and pathological conditions.

To further determine whether AT transplantation is involved in the increased glucose uptake observed in the HFD-induced diabetic limb ischemia mouse model, we performed glucose and insulin tolerance testing *in vivo*. These data revealed improved insulin sensitivity in HFD mice transplanted with normal WAT, as well as improved glucose tolerance in mice receiving BAT transplantation. The mechanism for this effect is unclear, but these findings suggest that transplantation of AT likely promotes skeletal muscle glucose uptake through an endocrine-related mechanism (Stanford et al., 2015). These endocrine effects are likely mediated by reduced TNF-a levels in ischemic muscles. In addition, increased IL-6 is linked to improved glucose metabolism after BAT transplantation (Stanford et al., 2013). The pathophysiologic mechanisms by which local transplanted adipose tissue influences the development of vascular disease remain to be determined. Our results showed that local transplantation of WAT has a pivotal role in improving blood perfusion and systemic metabolism in diabetic limb ischemia and that M2 macrophage activation is involved in the development of arteriogenesis. A limitation is the lack of relevant data on the association between WAT transplantation and glucose uptake into reperfused muscle in the present study. Future studies are needed to investigate whether the effect of AT transplantation on glucose homeostasis is related to glucose uptake in skeletal muscle during ischemia model. The results presented herein may provide a basis for the development of novel treatment options for diabetic or hypercholesterolemic PDA patients.

## Author contributions

All authors made substantial contributions to the conception and design of the various aspects of the prospective studies or to the acquisition, analysis or interpretation of data. All authors also contributed to drafting the article or revising it critically for important intellectual content and have given final approval of the version to be published. JW and RL are responsible for the integrity of this work as a whole, including the study design, access to data, and the decision to submit and publish the manuscript.

### Conflict of interest statement

The authors declare that the research was conducted in the absence of any commercial or financial relationships that could be construed as a potential conflict of interest. The reviewer GH and handling Editor declared their shared affiliation.

## Supplementary material

Supplemental Table 1 Sequences of primers used in the study.

Supplemental Table 2 Circulating hormone and lipid levels. Mice were sham-operated (Sham) or transplanted with WAT or BAT and studied 21 days after transplantation.

Supplemental Figure 1 Representative fluorescence images of ischemic adductor muscles. Mice were fed HFD for 14 weeks, followed by transplantation of WAT, BAT-derived from eGFP mice for 21 days. Scale bars, 100 μm.

Supplemental Figure 2 Total body weight in HFD-mice, followed by transplantation of WAT, BAT, and sham. Mice were fed HFD for 14 weeks, by 21 days after transplantation in all subject groups, there was no significant difference in the total weight.

Supplemental Figure 3 Macrophage quantification in white adipose tissue. **(A)** Representative images of macrophages as assessed by F4/80 Immunofluorescence in subcutaneous WAT of ND-WT and HFD-WT. Scale bars, 100 μm. **(B)** Quantification of anti-F4/80 positive-macrophage infiltration of subcutaneous WAT. ^*^*P* < 0.05 vs. ND-WT. ND: normal diet.

## References

[B1] Arnold L. Henry A. Poron F. Baba-Amer Y. van Rooijen N. Plonquet A.. (2007). Inflammatory monocytes recruited after skeletal muscle injury switch into antiinflammatory macrophages to support myogenesis. J. Exp. Med. 204, 1057–1069. 10.1084/jem.20070075 17485518 PMC2118577

[B2] Arras M. Ito W. D. Scholz D. Winkler B. Schaper J. Schaper W. (1998). Monocyte activation in angiogenesis and collateral growth in the rabbit hindlimb. J. Clin. Invest. 101, 40–50. 10.1172/JCI119877 9421464 PMC508538

[B3] Azuma K. Heilbronn L. K. Albu J. B. Smith S. R. Ravussin E. Kelley D. E.. (2007). Adipose tissue distribution in relation to insulin resistance in type 2 diabetes mellitus. Am. J. Physiol. Endocrinol. Metab. 293, E435–E442. 10.1152/ajpendo.00394.2006 17440034 PMC5684697

[B4] Barandier C. Montani J. P. Yang Z. (2005). Mature adipocytes and perivascular adipose tissue stimulate vascular smooth muscle cell proliferation: effects of aging and obesity. Am. J. Physiol. Heart Circ. Physiol. 289, H1807–H1813. 10.1152/ajpheart.01259.2004 16024563

[B5] Bourlier V. Zakaroff-Girard A. Miranville A. De Barros S. Maumus M. Sengenes C.. (2008). Remodeling phenotype of human subcutaneous adipose tissue macrophages. Circulation 117, 806–815. 10.1161/CIRCULATIONAHA.107.724096 18227385

[B6] Chawla A. Nguyen K. D. Goh Y. P. (2011). Macrophage-mediated inflammation in metabolic disease. Nat. Rev. Immunol. 11, 738–749. 10.1038/nri3071 21984069 PMC3383854

[B7] Cho C. H. Koh Y. J. Han J. Sung H. K. Jong Lee H. Morisada T.. (2007). Angiogenic role of LYVE-1-positive macrophages in adipose tissue. Circ. Res. 100, e47–e57. 10.1161/01.RES.0000259564.92792.93 17272806

[B8] Cnop M. Landchild M. J. Vidal J. Havel P. J. Knowles N. G. Carr D. R.. (2002). The concurrent accumulation of intra-abdominal and subcutaneous fat explains the association between insulin resistance and plasma leptin concentrations: distinct metabolic effects of two fat compartments. Diabetes 51, 1005–1015. 10.2337/diabetes.51.4.1005 11916919

[B9] Criqui M. H. Aboyans V. (2015). Epidemiology of peripheral artery disease. Circ. Res. 116, 1509–1526. 10.1161/CIRCRESAHA.116.303849 25908725

[B10] Fox C. S. Massaro J. M. Schlett C. L. Lehman S. J. Meigs J. B. O'Donnell C. J.. (2010). Periaortic fat deposition is associated with peripheral arterial disease: the Framingham heart study. Circ. Cardiovasc. Imaging. 3, 515–519. 10.1161/CIRCIMAGING.110.958884 20639302 PMC3060043

[B11] Fujisaka S. Usui I. Bukhari A. Ikutani M. Oya T. Kanatani Y.. (2009). Regulatory mechanisms for adipose tissue M1 and M2 macrophages in diet-induced obese mice. Diabetes 58, 2574–2582. 10.2337/db08-1475 19690061 PMC2768159

[B12] Gautier J. F. Mourier A. de Kerviler E. Tarentola A. Bigard A. X. Villette J. M.. (1998). Evaluation of abdominal fat distribution in noninsulin-dependent diabetes mellitus: relationship to insulin resistance. J. Clin. Endocrinol. Metab. 83, 1306–1311. 10.1210/jcem.83.4.4713 9543160

[B13] Gealekman O. Burkart A. Chouinard M. Nicoloro S. M. Straubhaar J. Corvera S. (2008). Enhanced angiogenesis in obesity and in response to PPARgamma activators through adipocyte VEGF and ANGPTL4 production. Am. J. Physiol. Endocrinol. Metab. 295, E1056–E1064. 10.1152/ajpendo.90345.2008 18728224 PMC2584813

[B14] Gealekman O. Guseva N. Hartigan C. Apotheker S. Gorgoglione M. Gurav K.. (2011). Depot-specific differences and insufficient subcutaneous adipose tissue angiogenesis in human obesity. Circulation 123, 186–194. 10.1161/CIRCULATIONAHA.110.970145 21200001 PMC3334340

[B15] Gordon S. Plüddemann A. Martinez Estrada F. (2014). Macrophage heterogeneity in tissues: phenotypic diversity and functions. Immunol. Rev. 262, 36–55. 10.1111/imr.12223 25319326 PMC4231239

[B16] Guglielmi V. Cardellini M. Cinti F. Corgosinho F. Cardolini I. D'Adamo M. (2015). Omental adipose tissue fibrosis and insulin resistance in severe obesity. Nutr Diabetes 10:e175 10.1038/nutd.2015.22 PMC4558556 26258766

[B17] Hazarika S. Dokun A. O. Li Y. Popel A. S. Kontos C. D. Annex B. H. (2007). Impaired angiogenesis after hindlimb ischemia in type 2 diabetes mellitus: differential regulation of vascular endothelial growth factor receptor 1 and soluble vascular endothelial growth factor receptor 1. Circ. Res. 101, 948–956. 10.1161/CIRCRESAHA.107.160630 17823371

[B18] Henrichot E. Juge-Aubry C. E. Pernin A. Pache J. C. Velebit V. Dayer J. M.. (2005). Production of chemokines by perivascular adipose tissue: a role in the pathogenesis of atherosclerosis? Arterioscler. Thromb. Vasc. Biol. 25, 2594–2599. 10.1161/01.ATV.0000188508.40052.35 16195477

[B19] Hocking S. L. Stewart R. L. Brandon A. E. Suryana E. Stuart E. Baldwin E. M.. (2015). Subcutaneous fat transplantation alleviates diet-induced glucose intolerance and inflammation in mice. Diabetologia 58, 1587–1600. 10.1007/s00125-015-3583-y 25899451

[B20] Kodelja V. Müller C. Tenorio S. Schebesch C. Orfanos C. E. Goerdt S. (1997). Differences in angiogenic potential of classically vs alternatively activated macrophages. Immunobiology 197, 478–493. 10.1016/S0171-2985(97)80080-0 9413747

[B21] Lumeng C. N. Bodzin J. L. Saltiel A. R. (2007a). Obesity induces a phenotypic switch in adipose tissue macrophage polarization. J. Clin. Invest. 117, 175–184. 10.1172/JCI29881 17200717 PMC1716210

[B22] Lumeng C. N. Deyoung S. M. Bodzin J. L. Saltiel A. R. (2007b). Increased inflammatory properties of adipose tissue macrophages recruited during diet-induced obesity. Diabetes 56, 16–23. 10.2337/db06-1076 17192460

[B23] Min S. Y. Kady J. Nam M. Rojas-Rodriguez R. Berkenwald A. Kim J. H.. (2016). Human 'brite/beige' adipocytes develop from capillary networks, and their implantation improvesmetabolic homeostasis in mice. Nat. Med. 22, 312–318. 10.1038/nm.4031 26808348 PMC4777633

[B24] Nakagami H. Maeda K. Morishita R. Iguchi S. Nishikawa T. Takami Y.. (2005). Novel autologous cell therapy in ischemic limb disease through growth factor secretion by cultured adipose tissue-derived stromal cells. Arterioscler. Thromb. Vasc. Biol. 25, 2542–2547. 10.1161/01.ATV.0000190701.92007.6d 16224047

[B25] Nishimura S. Manabe I. Nagasaki M. Hosoya Y. Yamashita H. Fujita H.. (2007). Adipogenesis in obesity requires close interplay between differentiating adipocytes, stromal cells, and blood vessels. Diabetes 56, 1517–1526. 10.2337/db06-1749 17389330

[B26] Nucera S. Biziato D. De Palma M. (2011). The interplay between macrophages and angiogenesis in development, tissue injury and regeneration. Int. J. Dev. Biol. 55, 495–503. 10.1387/ijdb.103227sn 21732273

[B27] Pasarica M. Sereda O. R. Redman L. M. Albarado D. C. Hymel D. T. (2009). Reduced adipose tissue oxygenation in human obesity: evidence for rarefaction, macrophage chemotaxis, and inflammation without an angiogenic response. Diabetes 58, 718–725. 10.2337/db08-1098 19074987 PMC2646071

[B28] Planas A. Clara A. Pou J. M. Vidal-Barraquer F. Gasol A. de Moner A.. (2001). Relationship of obesity distribution and peripheral arterial occlusive disease in elderly men. Int. J. Obes. Relat. Metab. Disord. 25, 1068–1070. 10.1038/sj.ijo.0801638 11443508

[B29] Schaper W. (2009). Collateral circulation: past and present. Basic Res. Cardiol. 104, 5–21. 10.1007/s00395-008-0760-x 19101749 PMC2755790

[B30] Simons M. Ware J. A. (2003). Therapeutic angiogenesis in cardiovascular disease. Nature Rev. Drug Discov. 2, 863–872. 10.1038/nrd1226 14668807

[B31] Stanford K. I. Middelbeek R. J. Townsend K. L. An D. Nygaard E. B. Hitchcox K. M. (2013). Brown adipose tissue regulates glucose homeostasis and insulin sensitivity. J. Clin. Invest. 23, 215–223. 10.1172/JCI62308 PMC3533266 23221344

[B32] Stanford K. I. Middelbeek R. J. Townsend K. L. Lee M. Y. Takahashi H. So K.. (2015). A novel role for subcutaneous adipose tissue in exercise-induced improvements in glucose homeostasis. Diabetes 64, 2002–2014. 10.2337/db14-0704 25605808 PMC4439563

[B33] Takeda Y. Costa S. Delamarre E. Roncal C. Leite de Oliveira R. Squadrito M. L.. (2011). Macrophage skewing by Phd2 haplodeficiency prevents ischaemia by inducing arteriogenesis. Nature 479, 122–126. 10.1038/nature10507 21983962 PMC4659699

[B34] Thomou T. Mori M. A. Dreyfuss J. M. Konishi M. Sakaguchi M. Wolfrum C.. (2007). Adipose-derived circulating miRNAs regulate gene expression in other tissues. Nature 542, 450–455. 10.1038/nature21365 28199304 PMC5330251

[B35] Tran K. V. Gealekman O. Frontini A. Zingaretti M. C. Morroni M. Giordano A.. (2012). The vascular endothelium of the adipose tissue gives rise to both white and brown fat cells. Cell Metab. 15, 222–229. 10.1016/j.cmet.2012.01.008 22326223 PMC3278718

[B36] Tran T. T. Yamamoto Y. Gesta S. Kahn C. R. (2008). Beneficial effects of subcutaneous fat transplantation on metabolism. Cell Metab. 7, 410–420. 10.1016/j.cmet.2008.04.00432 18460332 PMC3204870

[B37] Troidl C. Jung G. Troidl K. Hoffmann J. Mollmann H. Nef H.. (2013). The temporal and spatial distribution of macrophage subpopulations during arteriogenesis. Curr. Vasc. Pharmacol. 11, 5–12. 10.1016/j.cmet.2008.04.004 23391417

[B38] Wu J. Strawn T. L. Luo M. Wang L. Li R.. (2015). Plasminogen activator inhibitor-1 inhibits angiogenic signaling by uncoupling vascular endothelial growth factor receptor-2-αVβ3 integrin cross talk. Arterioscler. Thromb. Vasc. Biol. 35, 111–120. 10.1161/ATVBAHA.114.304554 25378411 PMC4270947

[B39] Xue J. Schmidt S. V. Sander J. Draffehn A. Krebs W. Quester I.. (2014). Transcriptome-based network analysis reveals a spectrum model of human macrophage activation. Immunity 40, 274–288. 10.1016/j.immuni.2014.01.00634 24530056 PMC3991396

