# Corrigendum: Transplantation of Normal Adipose Tissue Improves Blood Flow and Reduces Inflammation in High Fat Fed Mice With Hindlimb Ischemia

**DOI:** 10.3389/fphys.2018.00717

**Published:** 2018-06-05

**Authors:** Liyuan Chen, Lin Wang, Yongjie Li, Liqun Wuang, Yaofang Liu, Ningbo Pang, Yulin Luo, Jing He, Liping Zhang, Ni Chen, Rong Li, Jianbo Wu

**Affiliations:** ^1^Drug Discovery Research Center, Southwest Medical University, Luzhou, China; ^2^Laboratory for Cardiovascular Pharmacology of Department of Pharmacology, The School of Pharmacy, Southwest Medical University, Luzhou, China; ^3^Department of Gynaecology and Obstetrics, The Affiliated Hospital of Southwest Medical University, Luzhou, China; ^4^Dalton Cardiovascular Research Center, University of Missouri, Columbia, MO, United States

**Keywords:** arteriogenesis, inflammation, adipose tissue, blood perfusion, high-fat diet

During resubmission of the version of the manuscript, a previous version of Figure 3A was required by reviewer to merge images. We accidentally uploaded wrong images (Figure 3A, sham group; F8/40 staining) in the final version. The correct version of Figure 3A appears below. The authors sincerely apologize for the error. This error does not change the scientific conclusions of the research article.

**Figure 3:**
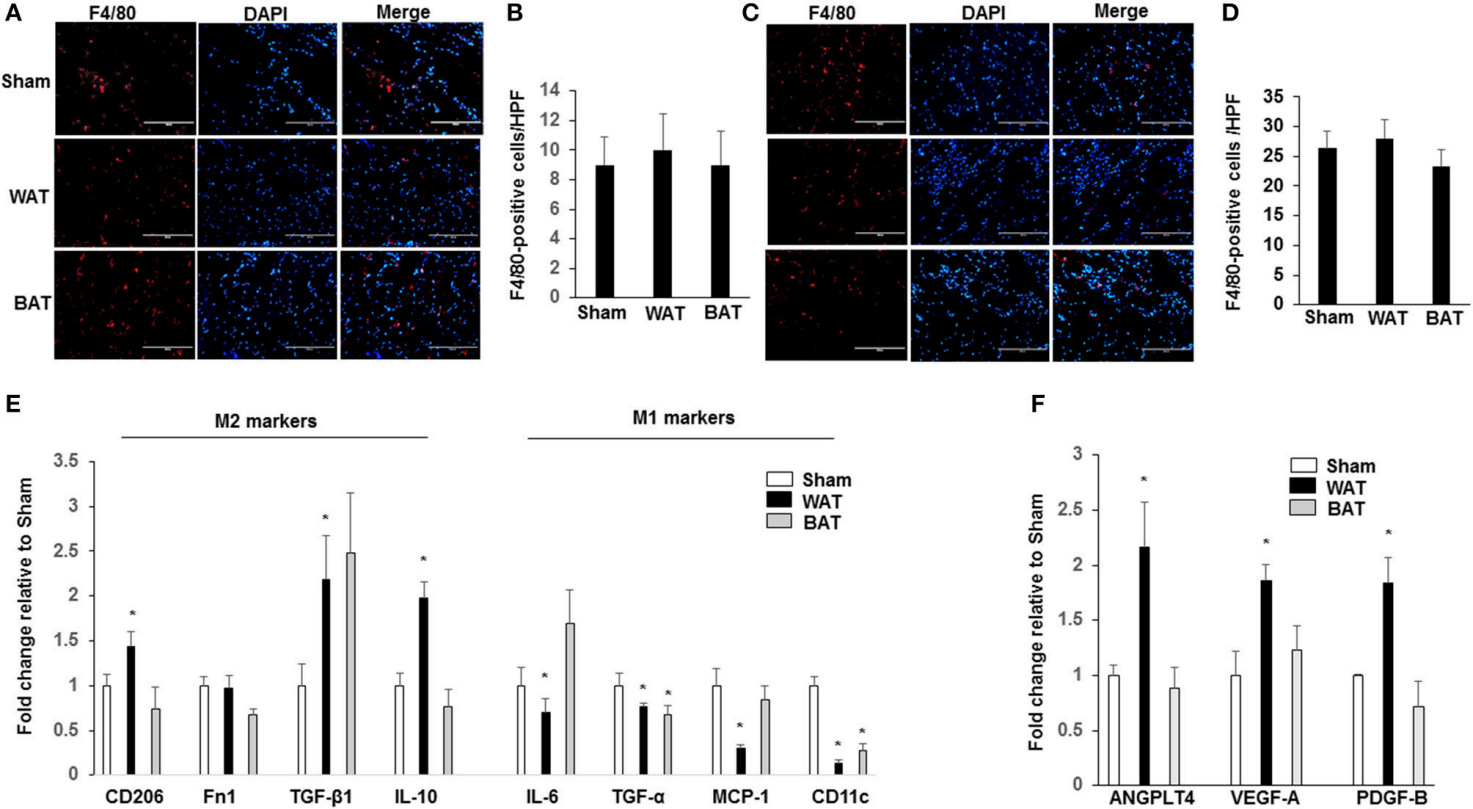
Macrophages display an M2-phenotype in ischemic muscles from transplanted WAT mice. Representative images of macrophages as assessed by F4/80 immunostaining, in ischemic adductor muscle **(A)** and gastrocnemius muscle **(C)** recovered 21 days after femoral artery interruption. Quantification of anti-F4/80 positive-macrophage infiltration of ischemic adductor muscle **(B)** and gastrocnemius muscle **(D)**. Scale bars, 100 μm. **(E)** The gene profile of the M1- and M2-macrophage phenotype by quantitative RT-PCR of ischemic adductor muscles 21 days after surgery. **(F)** The gene analysis of ANGPTL4, VEGF-A, and PDGF-B by quantitative RT-PCR of ischemic adductor muscles 21 days after surgery. All bars show Mean ± SEM. Data are mean of triplicate experiments and are expressed as fold-control. ^*^P < 0.05 toward sham-operated mice.

The original article has been updated.

## Conflict of interest statement

The authors declare that the research was conducted in the absence of any commercial or financial relationships that could be construed as a potential conflict of interest.

