# Supplementary material for: Transplantation of Normal Adipose Tissue Improves Blood Flow and Reduces Inflammation in High Fat Fed Mice With Hindlimb Ischemia
Source: Front Physiol. 2018 Mar 8;9:197. doi: 10.3389/fphys.2018.00197 (PMC5852102; doi:10.3389/fphys.2018.00197)
Supplement: Supplemental Table 1 — Sequences of primers used in the study. [file Table1.DOCX]

**Supplemental Table 1 Sequences of primers used in the study**

| CD11c | Forward | CACTCAGTGACTGCCCAAAA |
| --- | --- | --- |
|  | Reverse | CCTCAAGACAGGACATCGCT |
| IL-6 | Forward | CACATGTTCTCTGGGAAATCG |
|  | Reverse | TTGTATCTCTGGAAGTTTCAGATTGTT |
| TNF-α | Forward | ACGGCATGGATCTCAAAGAC |
|  | Reverse | AGATAGCAAATCGGCTGACG |
| MCP-1 | Forward | CTCTTTCCATTTTTGCATCAAGTTC |
|  | Reverse | CCCATCTTTAACCGATCTAGAGTCA |
| IL-10 | Forward | TGTCAAATTCATTCATGGCCT |
|  | Reverse | ATCGATTTCTCCCCTGTGAA |
| TGFβ1 | Forward | TGCTAATGGTGGACCGCAA |
|  | Reverse | CACTGCTTCCCGAATGTCTGA |
| VEGF-A | Forward | GCAGGCTGCTGTAACGATGAA |
|  | Reverse | TCACATCTGCTGTGCTGTAGGA |
| PDGF-B | Forward | CATCCGCTCCTTTGATGATCTT |
|  | Reverse | ATGAGCTTTCCAACTCGACTCC |
| CD206 | Forward | CATGGATGTTGATGGCTACTGGAG |
|  | Reverse | GTCTGTTCTGACTCTGGACACTTG |
| Fn1 | Forward | GGAGTGGCACTGTCAACCTC |
|  | Reverse | ACTGGATGGGGTGGGAAT |
| ANGPTL4 | Forward | GCATGGCTGCCTGTGGTAAC |
|  | Reverse | ATCTTGCTGTTTTGAGCCTTGA |
| GAPDH | Forward | TTCACCACCATGGAGAAGG |
|  | Reverse | CTCGTGGTTCACACCCATC |
