# Supplementary material for: Transplantation of Normal Adipose Tissue Improves Blood Flow and Reduces Inflammation in High Fat Fed Mice With Hindlimb Ischemia
Source: Front Physiol. 2018 Mar 8;9:197. doi: 10.3389/fphys.2018.00197 (PMC5852102; doi:10.3389/fphys.2018.00197)
Supplement: Supplemental Table 2 — Circulating hormone and lipid levels. Mice were sham-operated (Sham) or transplanted with WAT or BAT and studied 21 days after transplantation. [file Table2.DOCX]

Supplementary Table 2. Circulating hormone and lipid levels. Mice were sham-operated (Sham) or transplanted with WAT or BAT and studied 21 days after transplantation.

|  | **Sham** | **WAT** | **BAT** |
| --- | --- | --- | --- |
| Cholesterol (mmol/L) | 2.8 ± 1.23 | 2.5 ± 0.24*^#^ | 2.9 ± 0.11 |
| Triglycerides (mmol/L) | 0.27 ± 0.04 | 0.24 ± 0.03*^#^ | 0.33 ± 0.04 |
| Low-density lipoprotein(mmol/L) | 0.61 ± 0.06 | 0.52 ± 0.02*^#^ | 0.60 ± 0.04 |
| High-density lipoprotein(mmol/L) | 1.7 ± 0.09 | 1.6 ± 0.22*^#^ | 1.7 ± 0.06 |
| TNF-α (pg/mL) | 4.7 ± 0.9 | 4.6 ± 1.2^*#^ | 6.2 ± 1.7 |
| SDF-1α (pg/mL) | 145 ± 36 | 137 ± 13*^#^ | 138 ± 34 |

Values are mean ±SEM (n=9/group). *^,#^ p>0.05 Vs. Sham, BAT.
