# Supplementary figures and images for: Transplantation of Normal Adipose Tissue Improves Blood Flow and Reduces Inflammation in High Fat Fed Mice With Hindlimb Ischemia
Source: Front Physiol. 2018 Mar 8;9:197. doi: 10.3389/fphys.2018.00197 (PMC5852102; doi:10.3389/fphys.2018.00197)

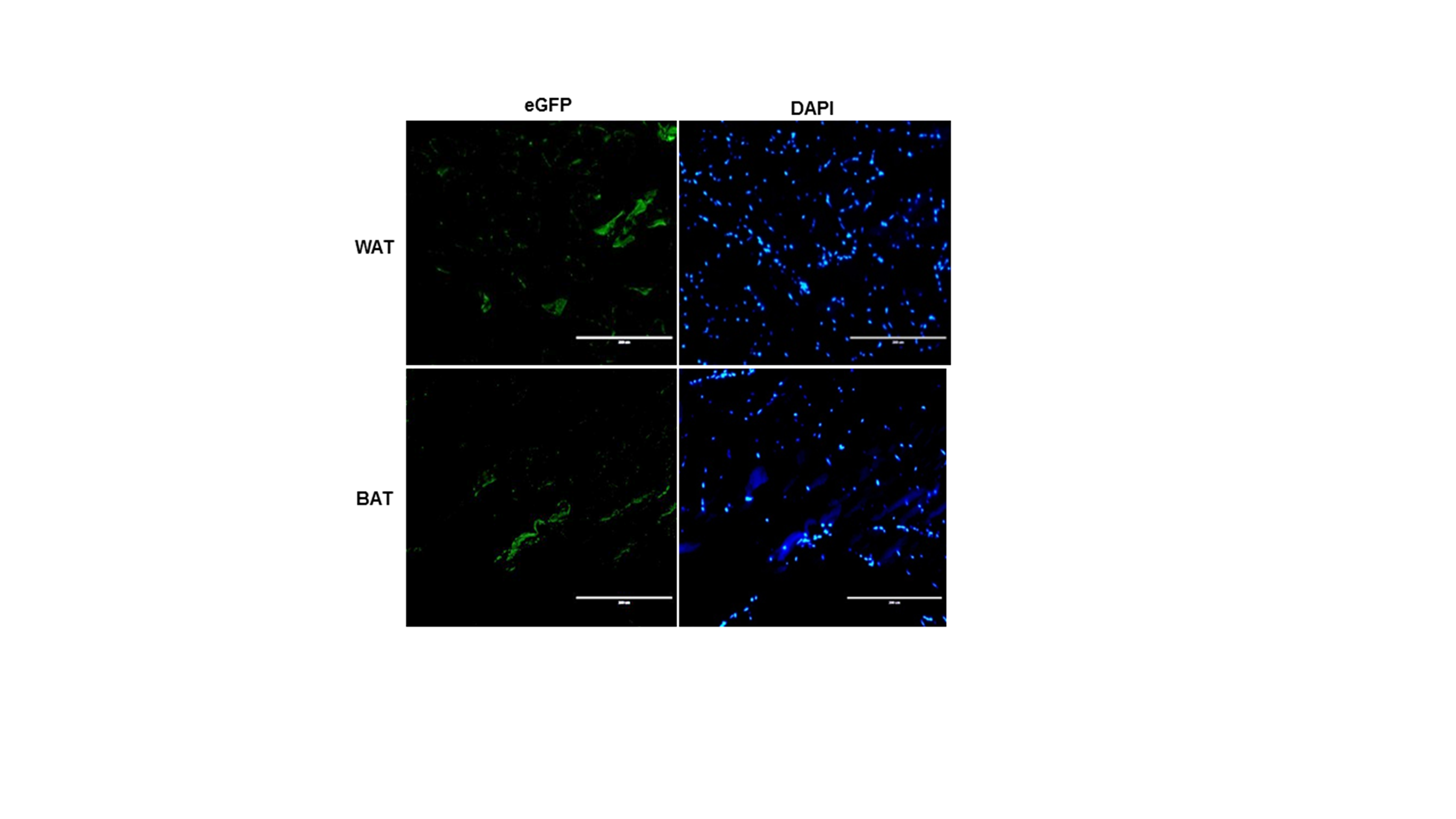

Supplement: Supplemental Figure 1 — Representative fluorescence images of ischemic adductor muscles. Mice were fed HFD for 14 weeks, followed by transplantation of WAT, BAT-derived from eGFP mice for 21 days. Scale bars, 100 μm. [file Image1.TIF]

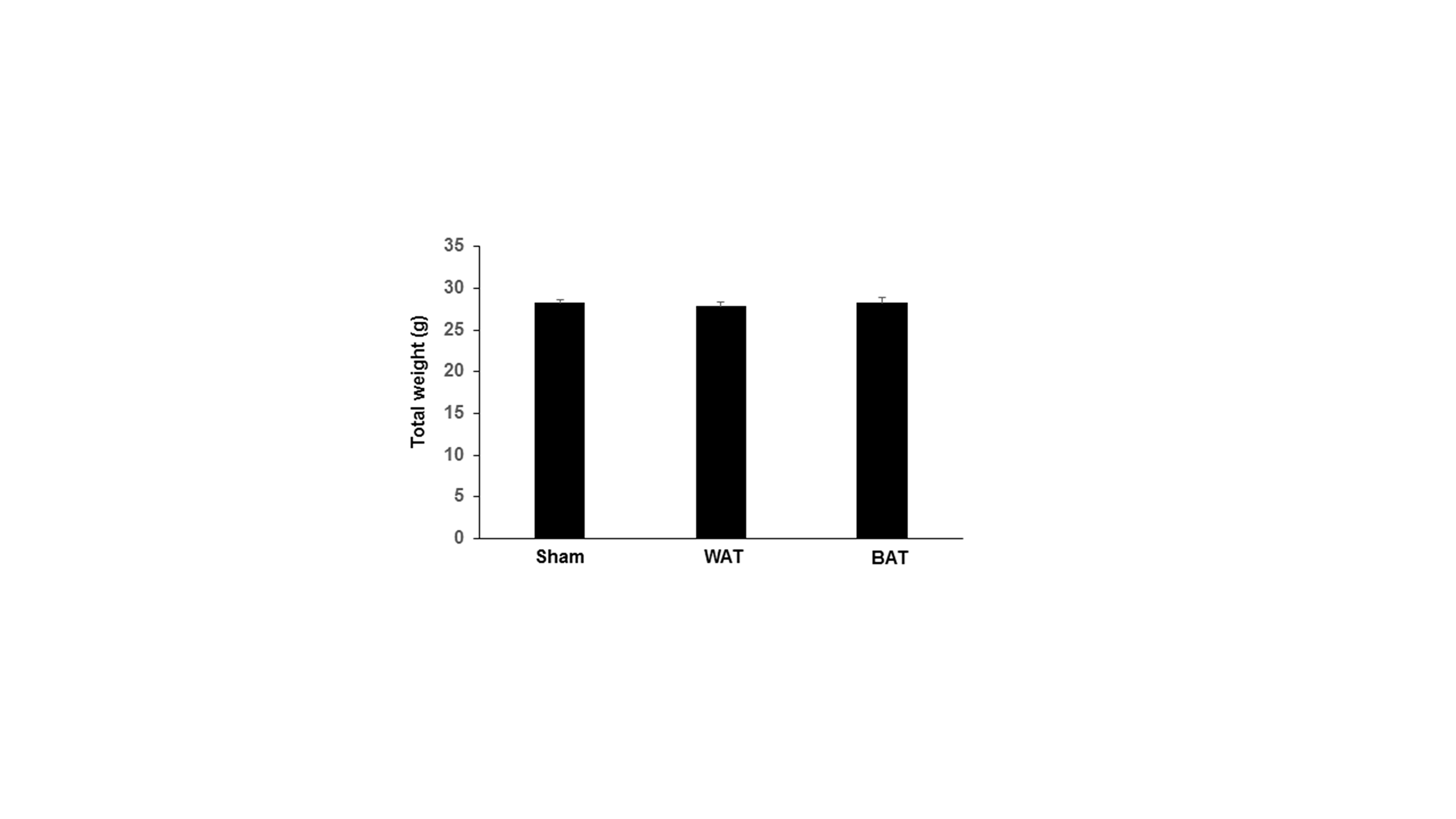

Supplement: Supplemental Figure 2 — Total body weight in HFD-mice, followed by transplantation of WAT, BAT, and sham. Mice were fed HFD for 14 weeks, by 21 days after transplantation in all subject groups, there was no significant difference in the total weight. [file Image2.TIF]

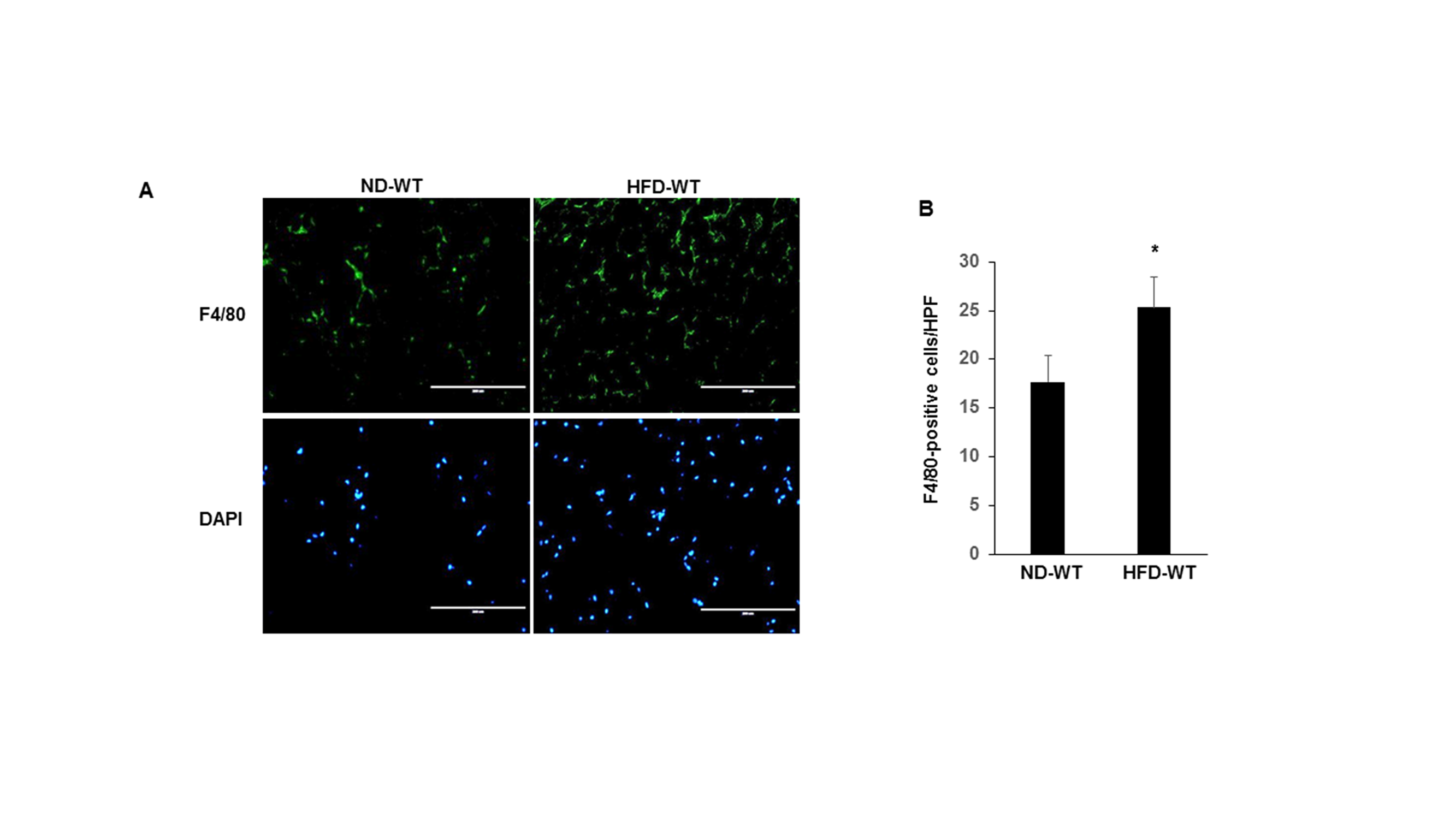

Supplement: Supplemental Figure 3 — Macrophage quantification in white adipose tissue. (A) Representative images of macrophages as assessed by F4/80 Immunofluorescence in subcutaneous WAT of ND-WT and HFD-WT. Scale bars, 100 μm. (B) Quantification of anti-F4/80 positive-macrophage infiltration of subcutaneous WAT. *P < 0.05 vs. ND-WT. ND: normal diet. [file Image3.TIF]
